# New Variants and Age Shift to High Fatality Groups Contribute to Severe Successive Waves in the 2009 Influenza Pandemic in Taiwan

**DOI:** 10.1371/journal.pone.0028288

**Published:** 2011-11-30

**Authors:** Ji-Rong Yang, Yuan-Pin Huang, Feng-Yee Chang, Li-Ching Hsu, Yu-Cheng Lin, Chun-Hui Su, Pei-Jer Chen, Ho-Sheng Wu, Ming-Tsan Liu

**Affiliations:** 1 Centers for Disease Control, Taipei, Taiwan, ROC; 2 College of Medicine, National Taiwan University, Taipei, Taiwan, ROC; 3 School of Medical Laboratory Science and Biotechnology, Taipei Medical University, Taipei, Taiwan, ROC; Hannover Medical School, Germany

## Abstract

Past influenza pandemics have been characterized by the signature feature of multiple waves. However, the reasons for multiple waves in a pandemic are not understood. Successive waves in the 2009 influenza pandemic, with a sharp increase in hospitalized and fatal cases, occurred in Taiwan during the winter of 2010. In this study, we sought to discover possible contributors to the multiple waves in this influenza pandemic. We conducted a large-scale analysis of 4703 isolates in an unbiased manner to monitor the emergence, dominance and replacement of various variants. Based on the data from influenza surveillance and epidemic curves of each variant clade, we defined virologically and temporally distinct waves of the 2009 pandemic in Taiwan from May 2009 to April 2011 as waves 1 and 2, an interwave period and wave 3. Except for wave 3, each wave was dominated by one distinct variant. In wave 3, three variants emerged and co-circulated, and formed distinct phylogenetic clades, based on the hemagglutinin (HA) genes and other segments. The severity of influenza was represented as the case fatality ratio (CFR) in the hospitalized cases. The CFRs in waves 1 and 2, the interwave period and wave 3 were 6.4%, 5.1%, 15.2% and 9.8%, respectively. The results highlight the association of virus evolution and variable influenza severity. Further analysis revealed that the major affected groups were shifted in the waves to older individuals, who had higher age-specific CFRs. The successive pandemic waves create challenges for the strategic preparedness of health authorities and make the pandemic uncertain and variable. Our findings indicate that the emergence of new variants and age shift to high fatality groups might contribute potentially to the occurrence of successive severe pandemic waves and offer insights into the adjustment of national responses to mitigate influenza pandemics.

## Introduction

Since an influenza outbreak caused by swine-origin influenza A (H1N1) viruses was detected initially in Mexico and USA during March and April 2009 [1], the viruses spread rapidly to an increasing number of countries. During the early stage of the 2009 pandemic, data from genetic analyses suggested that the influenza A (H1N1) 2009 viruses (termed “2009 H1N1 viruses” for convenience) had begun to evolve and diversified from April 1 to July 9, 2009 into at least 7 clades (clades 1–7) with spatial and geographic patterns [2], and the viruses in the early stage did not possess genomic signatures associated with high pathogenicity in the PB2, PB1-F2, HA and NS1 proteins [3]. Among the circulating viruses, the clade 7 viruses with a signature S220T substitution in the HA protein have spread more widely and become a globally major strain, and this dominated early in New York from April to July 2009 [4]. Some new variants derived from clade 7 were detected later in Australia, New Zealand, Singapore, Hong Kong and the United Kingdom [5], [6], [7], which raised the concern that the evolving viruses might be responsible for increased disease severity. The severity during the early 2009 pandemic was estimated to be less than that seen in the 1918 influenza pandemic and comparable to that seen in the 1957 pandemic [8]. The severity of the following autumn-winter pandemic wave in 2009–2010 remained mild and did not change, with mortality rates in the range from lower to slightly higher than that associated with seasonal influenza [9], [10], [11]. In the successive waves, increased severity was reported in Wales, UK and Wisconsin, USA [5], [12], [13], but data from New Zealand revealed that the overall impact of the second wave of the 2009 pandemic in 2010 was between one half and two thirds that of the first wave in 2009 [14]. The severity of the 2009 pandemic in the following years remains uncertain.

In Taiwan, the first case infected by 2009 H1N1 viruses was detected following imposed entry screening of a traveler from the USA on 20 May 2009 [15]. From July 2009, severe complicated influenza and death cases attributable to infection by 2009 H1N1 viruses occurred and began to be reported to the Centers for Disease Control, Taiwan. To clarify the relationship between the 2009 H1N1 viruses and disease severity during the successive waves, we analyzed comprehensively the evolution of 2009 H1N1 viruses isolated from May 2009 to April 2011 in Taiwan and defined virologically and temporally distinct waves of the 2009 pandemic, each of which was dominated by various variants. The case fatality ratio (CFR) in the hospitalization cases, representative of the severity, was found to increase in the successive waves and the age distribution of hospitalized cases was shifted to older groups, which had higher age-specific CFRs. The results reveal that virus changes and age shifts to the older groups with a high risk of death may contribute to the occurrence of successive waves in an influenza pandemic.

## Results

### Virus evolution of influenza A (H1N1) 2009 viruses in Taiwan from May 2009 to April 2011

**Figure 1:**
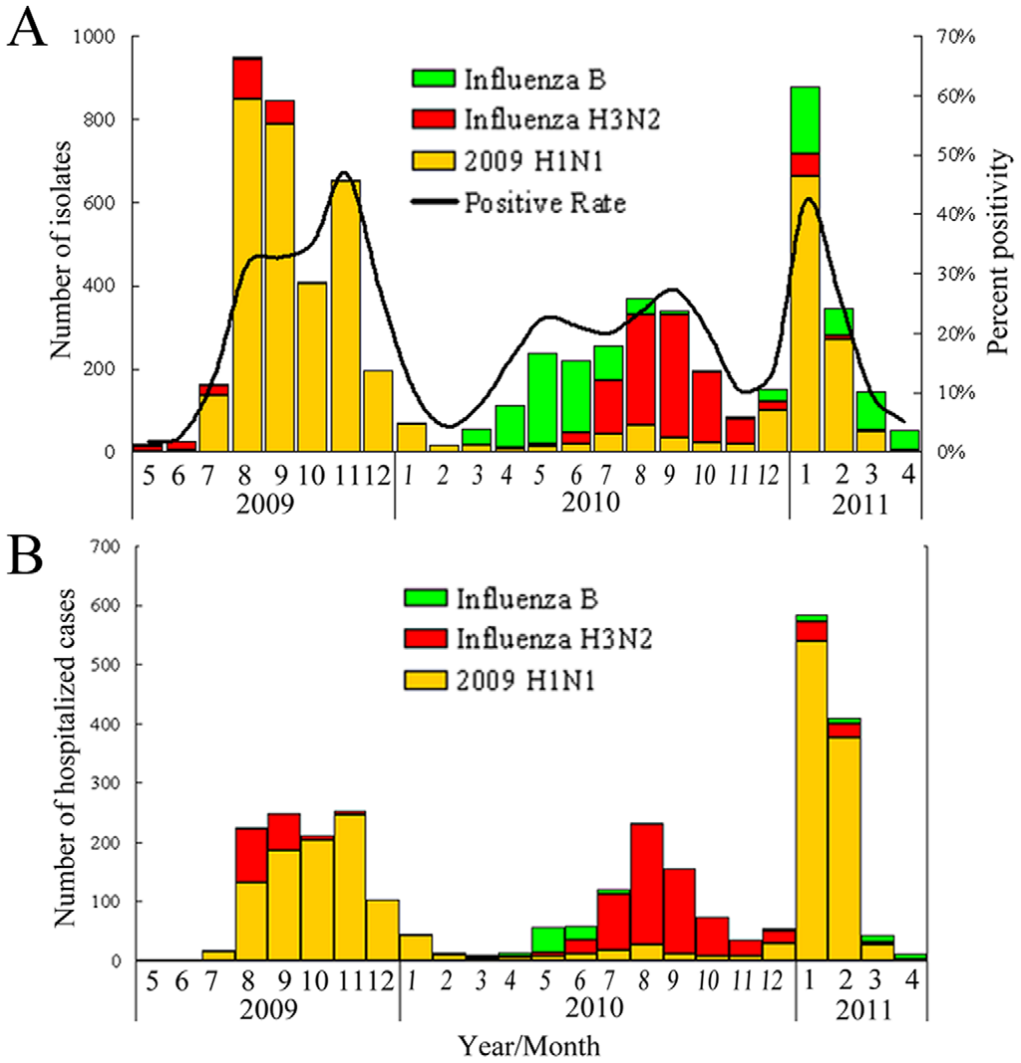
Monthly distribution of influenza isolates from (A) community outpatients and (B) hospitalized patients, confirmed in Taiwan from May 2009 to April 2011 by the laboratory-based surveillance network. Each virus is indicated by a bar of varying color and positivity rates of confirmed cases are included. The trends in both groups were similar. Influenza A (H1N1) 2009 viruses were the predominant subtype circulating from May 2009 to January 2010 and December 2010 to April 2011, while influenza A (H3N2) and influenza B viruses also were detected and co-circulated, especially from March to November in 2010. Influenza B viruses caused fewer hospitalized cases than the other two viruses.

**Figure 2:**
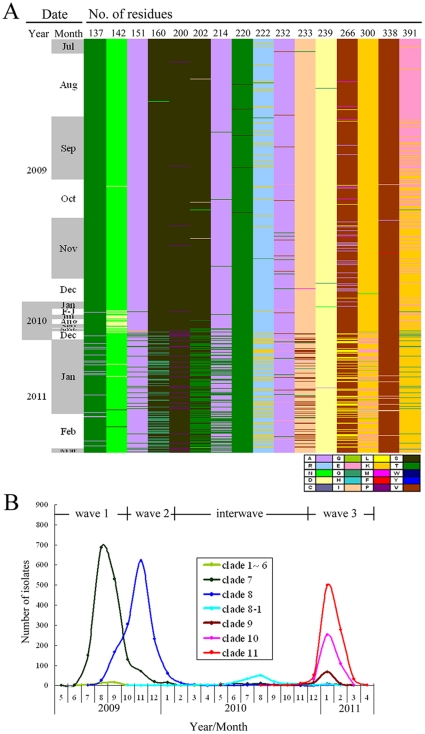
Evolution and emergence of influenza A (H1N1) 2009 viruses in Taiwan from May 2009 to April 2011. (A) Substitutions in the HA protein were visualized through a proteotyping map. Each column represents the indicated position of a specific amino acid, and these are shown by different colors, given in the key (single-letter abbreviations are used). Each row represents a single isolate and the 4703 analyzed isolates are displayed in the order of time of collection. (B) Various clade variants at different time periods were classified based on the HA genes. During the 2009 pandemic in Taiwan from May 2009 to April 2011, four periods were defined virologically and temporally as three major waves (waves 1, 2 and 3) and the interwave period. Each wave was dominated by one distinct variant, except for wave 3, in which three variants emerged and co-circulated. The major amino acid signatures of each clade were as follows: 220S for clade 1∼6, S220T-391E for clade 7, E391K for clade 8, N142D-E391K for clade 8-1, A151T-S200P-E391G for clade 9, R222K-V266L-K300E-E391K for clade 10, and S202T-E391K for clade 11 viruses.

**Figure 3:**
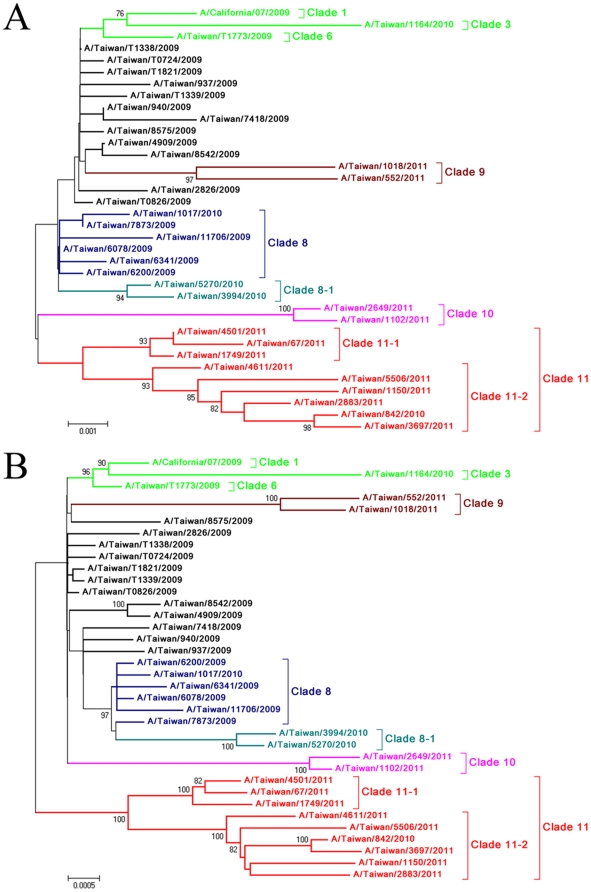
Phylogenetic relationships of the (A) HA and (B) PB2-PB1-PA-NP-NA-MP-NS concatenated sequences of influenza A (H1N1) 2009 viruses circulating from May 2009 to April 2011. The phylogenetic trees were constructed using the neighbor-joining method with 1000 bootstrap replications. Branch values of more than 70 are indicated. The genome sequences obtained from the NCBI database of the 6 early viral isolates collected before May 30, 2009 in Taiwan, A/Taiwan/T0724/2009, A/Taiwan/T0826/2009, A/Taiwan/T1338/2009, A/Taiwan/T1339/2009, A/Taiwan/T1773/2009, and A/Taiwan/T1821/2009, as well as the current vaccine strain, A/California/7/2009, are included as reference sequences. The classification of specific evolutionary clades is indicated.

Based on influenza laboratory surveillance from community and hospitalized cases in Taiwan, 2009 H1N1 viruses were prevalent from July 2009 to January 2010 and recurred with a sharp increase in hospitalized cases in December 2010. Other influenza epidemics, predominated by influenza B, accompanied by influenza A (H3N2) viruses, occurred from March to November 2010 (Fig. 1A, B). The virus distributions in community and hospitalized cases were similar except for influenza B viruses, which caused relatively fewer severe cases. During the two-year period, a total of 6451 cases infected by 2009 H1N1 viruses (4435 from community, and 2016 from hospitalized, cases) were diagnosed by real-time RT-PCR and/or virus culture. In order to monitor the detailed scenario of the time of introduction and evolutionary pattern of the newly emerging 2009 H1N1 viruses, 4703 available cultured viruses (3741 from community, and 962 from hospitalized cases) were selected and analyzed by sequencing their HA genes. We developed a protocol for analyzing the amino acid substitutions chronologically by directly visualizing the proteotyping map. After determining the amino acid sequences of the HA protein (residue positions 131–394) of the 4703 isolates, substitutions at specific positions with high entropy were plotted (Figure 2A). The proteotyping map revealed that, from May to early September of 2009, amino acid substitutions in the HA protein occurred in a sporadic and non-temporal, aggregative manner. Then, viruses carrying the substitution E391K emerged and became dominant in November. N142D was the second temporally aggregative substitution and occurred from February to November 2010, and was reversed thereafter (Fig. 2A). A larger number of amino acid substitutions began to be detected from December 2010, including T137A, A151T, S160G, S200P, S202T, A214T, R222K, I233V/G, V266L and K300E. The E/K391G substitution changed again and coexisted with the 391K population. These temporally aggregative substitutions can serve as signatures to define new variants and to differentiate clades in the phylogenetic analysis of HA sequences. Based on the proteotyping map and phylogenetic topology, these isolates were divided into various variants by HA clade and epidemic curves of the various variants were plotted (Fig. 2B). This revealed the clade prevalence during various periods in Taiwan. At the early stage during May to September 2009, most of the isolates had the significant signature S220T, which was previously defined as clade 7 by Nelson et al. [2]. Isolates with the E391K substitution, which were designated as clade 8 in this study, rapidly replaced clade 7 viruses and became dominant in November. During the period from February to November 2010, the dominant viruses changed again to clade 8–1, with both the E391K and N142D signatures, and circulated with low activity (Fig. 2B). In the influenza season of 2010–2011, from December 2010 another three new genetic variants, designated as clades 9, 10 and 11, emerged. The signatures of these viruses were 151T-200P-391G-526M, 14I-222K-233V/G-266L-300E-391K and 202T-391K-468N, respectively, while clade 11 could be further classified into clades 11-1 and 11-2 by the respective substitutions D114N and A214T (Fig. 3A and Table S1). The results showed that there was a total of three virus replacements, following the emergence of 2009 H1N1 viruses in Taiwan, and new genetic variants, with additional substitutions in the HA proteins, were formed by the last replacement. In addition to the analysis of HA genes, we also performed full-genomic sequencing of 29 representative isolates, selected from the various HA clade viruses (clade 1–6, 7, 8, 8–1, 9, 10, 11–1 and 11–2). The tree topologies of the concatenated PB2-PB1-PA-NP-NA-MP-NS sequences showed a similar pattern to that of the HA gene (Fig. 3B). This indicated that the newly emerging clade 9, 10 and 11 viruses were also phylogenetically distinct in the other genomic segments. Table S1 shows the clade specific substitution patterns of the 8 proteins encoded by the complete viral genome. In clades 8 and 8–1, amino acid changes in HA occurred simultaneously with those in the PB1, NP, NA and NS1 proteins. In clades 9, 10 and 11, HA substitutions paralleled those of the PB2, PB1, PA, NA, M1 and NS1 proteins (Table S1). This indicated that variants of 2009 H1N1 viruses emerging during the winter of 2010 had changed in multiple gene segments. The dynamic evolution of these viruses poses a potential threat of emerging pathogenic viruses.

### Varying influenza severity in the 2009 pandemic in Taiwan

**Figure 4:**
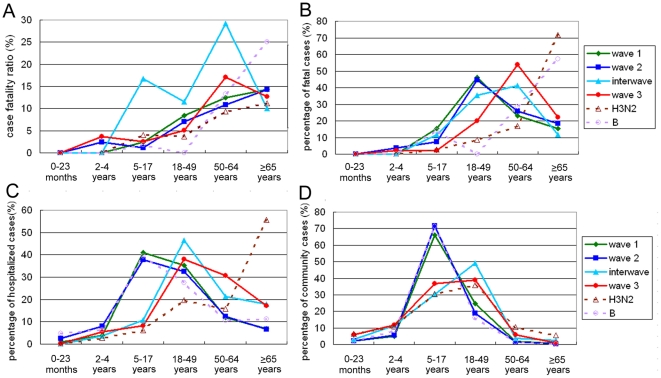
Age-specific case fatality ratio in hospitalized cases (A), age characteristics of percentage of fatality cases (B), hospitalized (C) and community (D) cases infected by 2009 H1N1 viruses in waves 1, 2, the interwave period and wave 3, as well as H3N2 and influenza B viruses. The number of fatality, hospitalized and community cases and their percentages for 2009 H1N1, H3N2 and influenza B viruses are described in Table 1 and 2.

To combine the data from influenza surveillance and epidemic curves of each clade variant, we defined virologically and temporally distinct waves of the 2009 pandemic from May 2009 to April 2011 in Taiwan as follows (Fig. 2B): The larger epidemic from May 2009 to January 2010 could be divided into the first two waves. Wave 1, dominated by clade 7 viruses, ran from May to early October 2009, followed by wave 2, dominated by clade 8 viruses, from late October 2009 to January 2010. The other peak, from December 2010 to April 2011, in which clades 9, 10 and 11 viruses co-circulated, was termed wave 3. Of note, the interwave period, dominated by specific clade 8-1 viruses, occurred from February to November 2010 during the period with a low activity of 2009 H1N1 viruses. Because the different waves were dominated by distinct clade variants, we wished to compare the influenza severity in these four time periods. The case fatality ratio (CFR) in the hospitalized cases was representative of the severity of influenza. The CFRs in four periods of waves 1 and 2, the interwave period and wave 3 were 6.4% (26/406), 5.1% (27/525), 15.2% (17/112) and 9.8% (95/972), respectively (Table 1). Among the three major waves of 2009 H1N1 viruses, the CFR of wave 3, which had an increasing number of fatal cases, was higher than those of the first two waves (Table 1; 9.8%, vs. 6.4%, p<0.05 and 5.1%, p<0.05). Of note, the highest CFR occurred in the interwave period (15.2%, p<0.05). For the co-circulating influenza A (H3N2) and influenza B viruses during this two-year period, the respective CFRs in the hospitalized patients were 8.7% (71/820) and 4.9% (7/142) (Table 2). The data reveal that the severity of the 2009 pandemic was lower than that of seasonal influenza (H3N2) at the early stage and increased in the following waves (p<0.05). To investigate why the CFRs increased in the successive waves, we compared in detail the age-specific CFRs in the hospitalized cases in various waves. The age-specific CFRs in hospitalized cases increased with age and were the highest in the group aged >65 years for H3N2, influenza B, as well as waves 1 and 2 of 2009 H1N1 infection (Fig. 4A). For the interwave period and wave 3 of 2009 H1N1 infection, individuals with the highest age-specific CFRs were adults aged 50–64 years (29.2%, 7/24 and 17.1%, 51/298, respectively), while those of the individuals aged >65 years remained as high as 10.0% (2/20 ) and 12.7% (21/166), respectively (Fig. 4A and Table 1). Regarding the percentage of fatal cases in each age group, young adults aged 18–49 years were the major group for 2009 H1N1 infection in waves 1 (46.1%, 12/26) and 2 (44.5%, 12/27), and this shifted to older adults aged 50–64 years (p<0.05) in the interwave period (41.2%, 7/17) and wave 3 (53.7%, 51/95), while individuals aged >65 years were the major group for H3N2 and influenza B infection (Fig. 4B and Table 1, 2). For the age characteristics of hospitalized and community cases, the major affected population for the 2009 H1N1 infection in waves 1 and 2 were school children aged 5–17 years, and this shifted to young adults aged 18–49 years in the interwave period and wave 3 (p<0.05). Of note, the percentages of the population older than 50 years who were hospitalized also increased to 39.3% (44/112) and 47.7% (464/972), respectively, in the interwave period and wave 3, accompanied by a dramatic increase in fatal cases (p<0.05). For H3N2 and influenza B infection, the major groups among hospitalized cases were individuals aged >65 years and school children, respectively, while those predominant among community cases were school children and young adults, respectively (Figure 4C, D and Table 1, 2). Based on these results, the age distribution of the infected populations in the successive waves of the 2009 H1N1 infection, including community and hospitalized cases, shifted significantly to older groups, who had higher age-specific CFRs and contributed to the increase of influenza severity during these stages of the 2009 pandemic in Taiwan.

**Table 1:** Cases infected by influenza A (H1N1) 2009 viruses in various periods in Taiwan from May 2009 to April 2011.

Age of patients	Wave 1				Wave 2				Interwave period				Wave 3			
	Community cases (%)	Hospitalized cases (%)	Fatal cases (%)	CFR*	Community cases (%)	Hospitalized cases (%)	Fatal cases (%)	CFR	Community cases (%)	Hospitalized cases (%)	Fatal cases (%)	CFR	Community cases (%)	Hospitalized cases (%)	Fatal cases (%)	CFR
0–23 months	41 (2.1)	4 (1.0)	0 (0)	0	25 (2.2)	14 (2.6)	0 (0)	0	8 (3.2)	0 (0)	0 (0)	0	63 (5.8)	3 (0.3)	0 (0)	0
2–4 years	95 (4.8)	16 (4.0)	0 (0)	0	63 (5.6)	42 (8.0)	1 (3.7)	2.4	29 (11.5)	4 (3.6)	0 (0)	0	126 (11.6)	54 (5.5)	2 (2.1)	3.7
5–17 years	1309 (66.1)	167 (41.1)	4 (15.4)	2.4	801 (71.6)	198 (37.7)	2 (7.4)	1.0	77 (30.4)	12 (10.7)	2 (11.8)	16.7	399 (36.9)	81 (8.3)	2 (2.1)	2.5
18–49 years	488 (24.6)	143 (35.2)	12 (46.1)	8.4	210 (18.8)	171 (32.6)	12 (44.5)	7.0	124 (49.0)	52 (46.4)	6 (35.3)	11.5	420 (38.8)	370 (38.1)	19 (20.0)	5.1
50–64 years	38 (1.9)	48 (11.8)	6 (23.1)	12.5	17 (1.5)	65 (12.4)	7 (25.9)	10.8	9 (3.6)	24 (21.4)	7 (41.2)	29.2	63 (5.8)	298 (30.7)	51 (53.7)	17.1
≥65 years	10 (0.5)	28 (6.9)	4 (15.4)	14.3	3 (0.3)	35 (6.7)	5 (18.5)	14.3	6 (2.4)	20 (17.9)	2 (11.7)	10.0	11 (1.0)	166 (17.1)	21 (22.1)	12.7
Total	1981 (100)	406 (100)	26 (100)	6.4	1119 (100)	525 (100)	27 (100)	5.1	253 (100)	112 (100)	17 (100)	15.2	1082 (100)	972 (100)	95 (100)	9.8

* **:** The case fatality ratio (CFR) in hospitalized cases was defined as the percentage of fatal cases among the total number of hospitalized influenza cases and was calculated as the total number of fatal cases divided by the total number of hospitalized cases.

**Table 2:** Cases infected by influenza A (H3N2) and influenza B viruses in Taiwan from May 2009 to April 2011.

Age of patients	Influenza A (H3N2)				Influenza B			
	Community cases (%)	Hospitalized cases (%)	Fatal cases (%)	CFR*	Community cases (%)	Hospitalized cases (%)	Fatal cases (%)	CFR
0–23 months	81 (6.4)	0 (0)	0 (0)	0	26 (2.3)	7 (4.9)	0 (0)	0
2–4 years	137 (10.9)	22 (2.7)	0 (0)	0	92 (8.3)	9 (6.3)	0 (0)	0
5–17 years	386 (30.7)	50 (6.1)	2 (2.8)	4	793 (71.2)	56 (39.4)	1 (14.3)	1.8
18–49 years	452 (35.9)	162 (19.8)	6 (8.5)	3.7	179 (16.1)	39 (27.5)	0 (0)	0
50–64 years	132 (10.5)	129 (15.7)	12 (16.9)	9.3	18 (1.6)	15 (10.6)	2 (28.6)	13.3
≥65 years	70 (5.6)	457 (55.7)	51 (71.8)	11.2	5 (0.4)	16 (11.3)	4 (57.1)	25.0
Total	1258 (100)	820 (100)	71 (100)	8.7	1113 (100)	142 (100)	7 (100)	4.9

* **:** The case fatality ratio (CFR) in hospitalized cases was defined as the percentage of fatal cases among the total number of hospitalized influenza cases and was calculated as the total number of fatal cases divided by the total number of hospitalized cases.

## Discussion

Past influenza pandemics, such as those caused by influenza A (H1N1) from 1918 to 1919, influenza A (H2N2) from 1957 to 1963 and influenza A (H3N2) from 1968 to 1970, have been characterized by several distinct features, including changes in the virus subtype, shifts of the highest death rates to younger populations, multiple waves, higher transmissibility than seasonal influenza, and varying impacts in different geographic regions [16]. These factors, especially information on virus evolution and disease severity during the continuous pandemic waves, were all crucial for evaluating the impact of the disease and for consideration of influenza response plans. For the 2009 pandemic, until now, the features of multiple waves remained unclear. The estimated severity indicated by the CFRs in hospitalized cases was 7% during April to June 2009 in the USA [17] and 4.1–8% in the USA, Norway and Austria in the following fall and winter [11], [18], [19]. In the Southern Hemisphere, the CFRs in hospitalized patients in 2009 (the first wave) varied between 2.4% and 7.6% in various studies [20]. In Taiwan, the CFR of the hospitalized cases calculated from July 2009 to January 2010 in this study was 5.7% (53/931), while those of the influenza A (H3N2) and influenza B viruses were 8.6% (71/820) and 4.9% (7/142), respectively. The data revealed that severity of 2009 H1N1 illness in the early stage was milder than that of seasonal influenza A (H3N2) viruses. However, in the successive wave from December 2010, the new genetic variants of clades 9, 10 and 11 viruses emerged with an increase in CFRs from 6.4% and 5.1% to 9.8% (p<0.05), indicating that successive severe waves of the 2009 pandemic occurred in Taiwan. Although the reasons for increased severity in successive waves were unclear, they were likely to include virus changes, seasonality, medical measures and the overall immunity of the population [16]. In this study, these factors also were considered. First, the increase in fatality between the waves of a pandemic was likely to be attributable to the generation and emergence of mutated viruses, with increased pathogenicity and greater adaptation to the human host, while we had observed that various variants of 2009 H1N1 viruses were dominant during different periods of the pandemic and associated with varying fatality in hospitalized patients. Genetic mutations and reassortments have been reported potentially to enhance the virulence of 2009 H1N1 viruses [21], [22], [23]. In Taiwan, the genome signatures of the evolving 2009 H1N1 viruses in successive waves were identified in this study, including T257A in PB1, E391K in HA and M93I in the NS1 protein of clade 8 viruses in wave 2; A652V in PB1, N142D in HA, K400R-K452R in NP, and M15I-N189S in NA of clade 8-1 in the interwave period; V225I-V511I-V584I-V667I in PB2, R211K-I435V in PB1, D479E in PA, A151T-S200P-E391G-R526M in HA, I389V-V394I in NA, and E55Q in NS1 of clade 9; A221S in PB2, V113A-K386R in PB1, V14I in PA, T14I-R222K-I233V/G-V266L-K300E in HA, S299A-I374V in NA, and P162L in NS1 of clade 10; V344M-V354L in PB2, N321K in PA, S202T-S468N in HA, V241I-N369K in NA and V80I in M1 of clade 11 (additional N456S in PB2, I330V in PA, and D114N in HA of clade 11-1, as well as I397M in PB1, A343T in PA, A214T in HA, N44S in NA and L90I in NS1 of clade 11-2) in wave 3 (Table S1). Among these HA mutations, N142D was located in the known antigenic Sa site, and R222K was located in the antigenic Ca site [24]. The mutations, S200P, A214T and I233V, near receptor binding sites may affect the interaction of HA with its receptor [25], [26]. Although only few of these residues had been reported [27], [28], their undetermined effects on virus pathogenicity may be significant. Another important amino acid substitution, D239G, was analyzed and compared in different waves; this is known to cause a shift to a dual α2–3/α2–6-sialic acid linkage specificity, allowing the mutant protein to bind to both human and avian receptors [29] and has reportedly been associated with severe cases [30]. In our study, the respective percentages of this D239G substitution in 2009 H1N1 viruses from hospitalized cases during waves 1 and 2, the interwave period and wave 3 were 1.9% (4/210), 1.5% (4/276), 7.9% (3/38) and 1.4% (6/438), while those in community cases were 0.3% (4/1597), 0% (0/977), 0.5% (1/190) and 0.1% (1/977), respectively. The percentages of viruses harboring 239G were in the ratio of 6.3–15 between those from hospitalized and community cases during the three major waves (1.9% vs. 0.3%; 1.5% vs. 0%; 1.4% vs. 0.1%, respectively). Of note, a dramatic percentage increase (7.9% of hospitalized vs. 0.5% of community cases, p<0.05) in the interwave period, accompanied by the highest CFR in hospitalized cases, also was observed. The occurrence of this substitution was not clade-specific, but these data could highlight the important impact of virus changes on influenza severity in future waves and it was therefore essential for continuous surveillance of the trends of virus evolution. The second factor, seasonality of various waves, was analysed. In Taiwan, wave 1 and the interwave period were outside the regular influenza season. Waves 2 and 3 were in the winter influenza season, although wave 2 was two months earlier than the usual timing. As cold temperature and low humidity have been reported to enhance influenza transmission in an animal model [31], the effect of seasonality on the severity of various waves of influenza was not observed in this study. The other factors, medical measures and overall immunity of the population, such as antiviral medication and vaccine administration, were discussed. During the two-year period, only few sporadic 2009 H1N1 viruses from cases after drug-treatment were found to carry the substitution H275Y in the NA protein, conferring resistance to oseltamivir [32]. The policy of use of government-funded antiviral agents, which aimed to decrease the spread of viruses and to minimize the occurrence of severe cases, is consistent and antiviral agents were prescribed for cases from cluster outbreaks and of reported severity, and extended to the patients who presented danger signs of developing severe disease during the peak of influenza activity. Therefore, the consistent policy of antiviral agents in Taiwan did not seem to involve in the variety of influenza severity in various waves. Finally, for overall immunity of the population, which was attributed to pre-exposure to infection and vaccination, was considered. Serologic data from previous studies at the early stage of the 2009 pandemic suggested that a higher proportion of persons aged above 60 years may have pre-existing immunity to the 2009 H1N1 viruses due to past infection [33]. It was also shown that the major population of hospitalized and fatal cases in the 2009 pandemic was younger than those commonly seen with seasonal influenza [34]. In our study, the attack rates of 2009 H1N1 viruses showed that school children aged 5-17 years were also the major affected targets of this virus in waves 1 and 2, which differed from those of influenza A (H3N2) viruses (Table 1 and 2; p<0.05). During the interwave period and wave 3, the predominant infected cases shifted to young adults and the percentages of school children in community and hospitalized cases decreased from 68.1% (2110/3100) and 39.2% (365/931) to 35.7% (476/1335) and 8.6% (93/1084), respectively. Of note, the cumulative percentage of hospitalized individuals aged >50 years increased from 18.9% (176/931) to 46.9% (508/1084), accompanied by an increasing number of fatal cases (p<0.05). These data suggested that the age shifts may result from the possible protection of infection-acquired immunity in the younger population after the early waves, following a higher attack rate at the beginning of the 2009 pandemic. Another possible effect may be attributed to influenza vaccination. In Taiwan, the vaccine coverage rates of populations, who had received at least one dose of H1N1 vaccine from November 2009 to March 2010, reached 22%, including 29%, 72%, and 11% of persons aged 6 months to 6 years, 7–18 years, and above 19 years, respectively [35], while the cumulative percentage of those who had received at least one dose of H1N1 vaccine from October 2010 to May 2011, reached 12.6%, including 25.8%, 64.4% and 37.3% of persons aged 6 months to 6 years, 7–12 years,and older than 65 years, respectively (data not shown). Individuals aged 13–64 years were not included in the government-funded vaccination program in 2010–2011 influenza season. This showed that the school children had the highest vaccination rate and adults aged 18–64 were the shortfall in influenza vaccination and the age-specific vaccine coverage seemed to contribute to the shift of age to older groups. In this study, we found that people aged above 50 years were the population with the highest age-specific CFR during the 2009 pandemic in Taiwan. This was similar to the scenario of the past influenza illness and data from the early 2009 pandemic, which showed that persons aged above 50 years had the highest rates of mortality once hospitalized [7], [19], [34], [36], [37]. The shift to older groups, who had relatively higher age-specific CFRs, may have contributed to the increased influenza severity in the successive waves of the 2009 pandemic in Taiwan.

Several signature features of the 2009 pandemic were observed in our study: emergence and replacement of the genetic variants, variable severity, and the targeted age shift to older groups. These factors of virus changes, seasonality, medical measures and the overall immunity of the population, which constitute unique features of an influenza pandemic, are complicated and variable. We found that both virus changes and age shifts to the older groups with high risks of fatality may be important factors to explain the increased severity during successive waves of an influenza pandemic.

## Materials and Methods

### Collection of clinical specimens and virus isolates

Clinical specimens from outpatients with influenza-like illnesses in communities (community cases, represented as mild cases) and hospitalized patients who developed severe complications (hospitalized cases, represented as severe cases) were collected and transported to the laboratories of the influenza surveillance network in Taiwan, which is coordinated by the Centers for Disease Control (Taiwan CDC), for influenza diagnosis using virus culture or/and real-time RT-PCR [15], [38]. For hospitalized cases in Taiwan, influenza with severe complications is a notifiable disease and patients who match one of the following criteria defined by clinical symptoms should be reported and their clinical specimens collected: (1) pulmonary complications (2) neurological complications (3) myocarditis or pericarditis (4) invasive bacterial infection (5) others: patients who have no above symptoms but need treatment in an intensive care unit. When performing diagnosis, both molecular analysis and direct virus isolation by cell culture were conducted and all of the influenza isolates from positive cases were transported to the Taiwan CDC for further characterization. Viral RNA extraction from clinical specimens, as well as influenza identification and subtyping, and the nucleotide sequences of viral genes were determined by conventional RT-PCR and sequencing and were processed using methods described previously [15], [32].

### Proteotyping map and epidemic curves of various 2009 H1N1 variants in Taiwan from 2009 to 2011

A proteotyping map of the HA gene was constructed from the putative amino acid sequences. For analysis, multiple alignments were made of the amino acid sequences. The positions with high entropy were then chosen and indicated on the map. Each type of amino acid was represented by a color. On the map, the amino acid residues of each virus were presented on the X axis and each virus was ordered according to the collection date on the Y axis. Based on the pattern of amino acid substitutions, we classified the virus isolates into various groups and the epidemic curves of 2009 H1N1 variants in Taiwan were plotted.

### Complete genome analysis of the 2009 H1N1 viruses

To investigate in detail the molecular phylogenies and genetic diversities of the 2009 H1N1 viruses circulating from May 2009 to April 2011 in Taiwan, full-genome sequences of the 29 representative viruses selected were determined. Sequences obtained from the NCBI database of the 6 early viral isolates collected before May 30, 2009 in Taiwan, A/Taiwan/T0724/2009, A/Taiwan/T0826/2009, A/Taiwan/T1338/2009, A/Taiwan/T1339/2009, A/Taiwan/T1773/2009, and A/Taiwan/T1821/2009, as well as the current vaccine strain, A/California/7/2009, were included as reference strains. The analyzed phylogenies were designated from clade 1–6 to clade 11 with reference to a previous report [2]. Multiple sequence alignments, protein translation and phylogenetic analysis were performed on the basis of nucleotide sequences using the software MEGA4 and BioEdit (http://www.mbio.ncsu.edu/BioEdit/bioedit.html). Phylogenetic trees were constructed by the neighbor-joining method and 1,000 bootstrap replications were performed to evaluate the robustness.

### Case fatality ratio

The case fatality ratio of hospitalized case was calculated as the total number of fatal cases divided by the total number of hospitalized cases. The age-specific CFRs were calculated as the number of fatal cases in a specific age group divided by the total number of hospitalized cases within that group.

### Statistical Analysis

Descriptive statistics were calculated to describe data from the influenza seasons. We used Microsoft Excel (2003) to produce figures and calculate descriptive statistics such as mean, range, and percentage. The chi-square test was performed using OpenEpi Version 2.3.1 (Dean AG, Sullivan KM, Soe MM. OpenEpi: Open Source Epidemiologic Statistics for Public Health, Version 2.3.1. www.OpenEpi.com, updated 2011/23/06, accessed 2011/10/19).

### Sequences information

Nucleotide sequences of influenza viruses in this study have been submitted to GenBank and their accession numbers are from JN187125-JN187356.

## Supporting Information

Table S1 **Genome signatures of the evolving influenza A (H1N1) 2009 viruses during various periods of the 2009 pandemic in Taiwan.**
(DOC)
