# Supplementary material for: New Variants and Age Shift to High Fatality Groups Contribute to Severe Successive Waves in the 2009 Influenza Pandemic in Taiwan
Source: PLoS One. 2011 Nov 30;6(11):e28288. doi: 10.1371/journal.pone.0028288 (PMC3227656; doi:10.1371/journal.pone.0028288)
Supplement: Table S1 — Genome signatures of the evolving influenza A (H1N1) 2009 viruses during various periods of the 2009 pandemic in Taiwan. (DOC) [file pone.0028288.s001.doc]

**Table S1.** **Genome signatures of the evolving influenza A (H1N1) 2009 viruses during various periods of the 2009 pandemic in Taiwan**

W1: wave 1, W2: wave 2, IW: interwave period, W3: wave 3
